# Supplementary material for: Novel Driver Strength Index highlights important cancer genes in TCGA PanCanAtlas patients
Source: PeerJ. 2022 Aug 11;10:e13860. doi: 10.7717/peerj.13860 (PMC9375969; doi:10.7717/peerj.13860)
Supplement: Supplemental Information 2 [file peerj-10-13860-s002.zip › COHORTS/genes plots/stage/2021_8_16_14_9_distribution_stage_I.pdf]

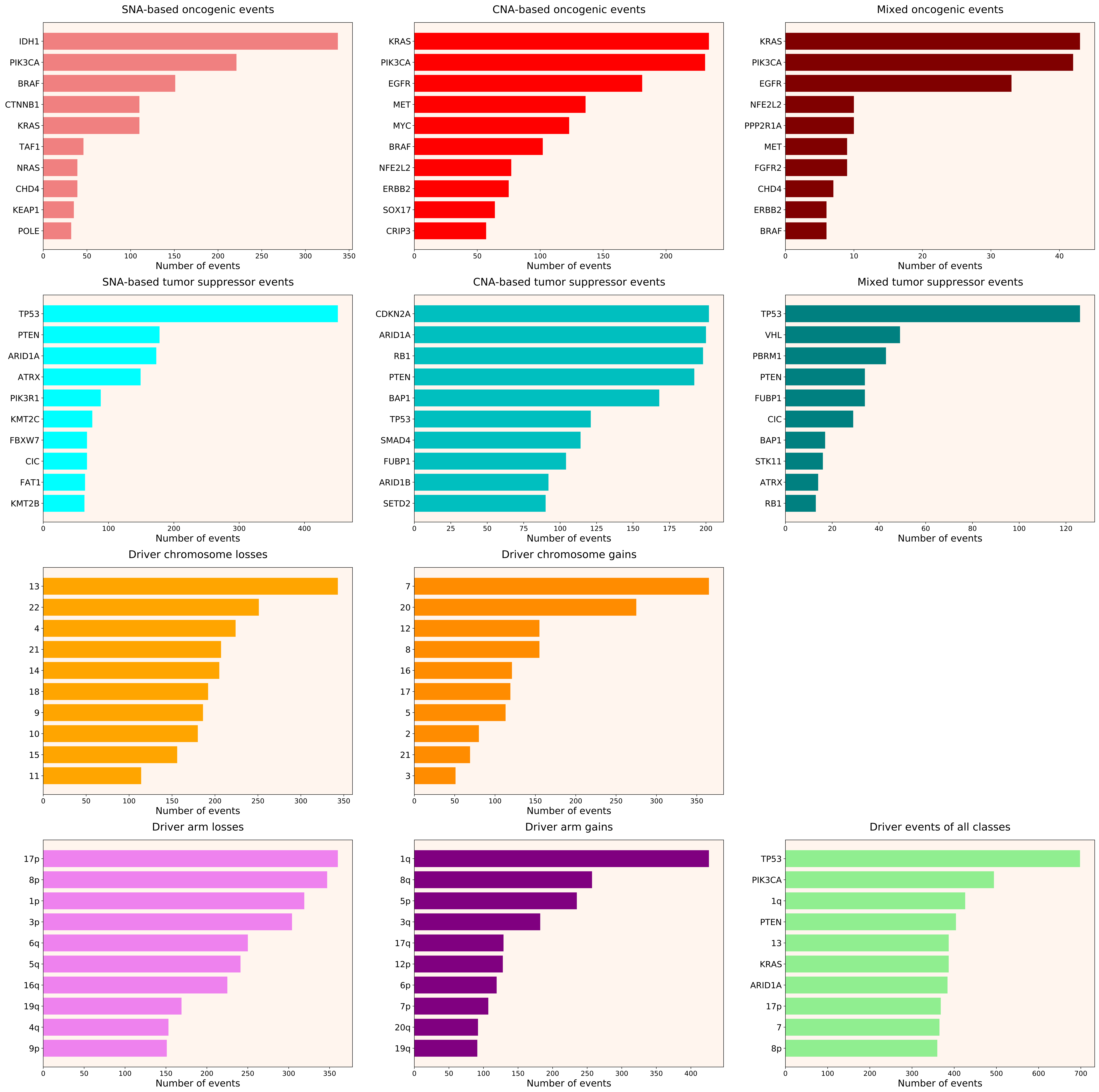
SNA-based oncogenic events
IDH1
PIK3CA
BRAF
CTNNB1
KRAS
TAF1
NRAS
CHD4
KEAP1
POLE
0 50 100 150 200 250 300 350
Number of events
CNA-based oncogenic events
KRAS
PIK3CA
EGFR
MET
MYC
BRAF
NFE2L2
ERBB2
SOX17
CRIP3
0 50 100 150 200
Number of events
Mixed oncogenic events
KRAS
PIK3CA
EGFR
NFE2L2
PPP2R1A
MET
FGFR2
CHD4
ERBB2
BRAF
0 10 20 30 40
Number of events
SNA-based tumor suppressor events
TP53
PTEN
ARID1A
ATRX
PIK3R1
KMT2C
FBXW7
CIC
FAT1
KMT2B
0 100 200 300 400
Number of events
CNA-based tumor suppressor events
CDKN2A
ARID1A
RB1
PTEN
BAP1
TP53
SMAD4
FUBP1
ARID1B
SETD2
0 25 50 75 100 125 150 175 200
Number of events
Mixed tumor suppressor events
TP53
VHL
PBRM1
PTEN
FUBP1
CIC
BAP1
STK11
ATRX
RB1
0 20 40 60 80 100 120
Number of events
Driver chromosome losses
13
22
4
21
14
18
9
10
15
11
0 50 100 150 200 250 300 350
Number of events
Driver chromosome gains
7
20
12
8
16
17
5
2
21
3
0 50 100 150 200 250 300 350
Number of events
Driver arm losses
17p
8p
1p
3p
6q
5q
16q
19q
4q
9p
0 50 100 150 200 250 300 350
Number of events
Driver arm gains
1q
8q
5p
3q
17q
12p
6p
7p
20q
19q
0 50 100 150 200 250 300 350 400
Number of events
Driver events of all classes
TP53
PIK3CA
1q
PTEN
13
KRAS
ARID1A
17p
7
8p
0 100 200 300 400 500 600 700
Number of events
